# Gas-Foamed Scaffold Gradients for CombinatorialScreening in 3D

**DOI:** 10.3390/jfb3010173

**Published:** 2012-03-07

**Authors:** Kaushik Chatterjee, Alison M. Kraigsley, Durgadas Bolikal, Joachim Kohn, Carl G. Simon

**Affiliations:** 1 Polymers Division, National Institute of Standards & Technology, 100 Bureau Drive, Gaithersburg, MD 20899, USA; E-Mail: alison.kraigsley@nist.gov; 2 Department of Materials Engineering, Indian Institute of Science, Bangalore 560012, India; E-Mail: kchatterjee@materials.iisc.ernet.in; 3 New Jersey Center for Biomaterials, Rutgers University, Piscataway, NJ 08854, USA;E-Mails: bolikal@biology.rutgers.edu (D.B.); kohn@rutgers.edu (J.K.)

**Keywords:** combinatorial screening, polymer, scaffold, tissue engineering

## Abstract

Current methods for screening cell-material interactions typically utilize a two-dimensional (2D) culture format where cells are cultured on flat surfaces. However, there is a need for combinatorial and high-throughput screening methods to systematically screen cell-biomaterial interactions in three-dimensional (3D) tissue scaffolds for tissue engineering. Previously, we developed a two-syringe pump approach for making 3D scaffold gradients for use in combinatorial screening of salt-leached scaffolds. Herein, we demonstrate that the two-syringe pump approach can also be used to create scaffold gradients using a gas-foaming approach. Macroporous foams prepared by a gas-foaming technique are commonly used for fabrication of tissue engineering scaffolds due to their high interconnectivity and good mechanical properties. Gas-foamed scaffold gradient libraries were fabricated from two biodegradable tyrosine-derived polycarbonates: poly(desaminotyrosyl-tyrosine ethyl ester carbonate) (pDTEc) and poly(desaminotyrosyl-tyrosine octyl ester carbonate) (pDTOc). The composition of the libraries was assessed with Fourier transform infrared spectroscopy (FTIR) and showed that pDTEc/pDTOc gas-foamed scaffold gradients could be repeatably fabricated. Scanning electron microscopy showed that scaffold morphology was similar between the pDTEc-rich ends and the pDTOc-rich ends of the gradient. These results introduce a method for fabricating gas-foamed polymer scaffold gradients that can be used for combinatorial screening of cell-material interactions in 3D.

## 1. Introduction

Combinatorial and high-throughput technologies are being applied for discovery in tissue engineering research to systematically screen cell-biomaterial interactions [1,2]. In current methods, material surfaces are prepared either as a continuous gradient or an array library where a property of the biomaterial is systematically varied [3,4,5,6,7]. Cells are seeded on the biomaterial libraries and optimal properties are determined through measurement of the desired cellular responses. For example, cell response to polymer blends was measured using gradient scaffolds prepared from poly(ε-caprolactone) (PCL) and poly(D,L)lactic acid (PDLLA) to identify an optimal composition for osteoblast proliferation and differentiation [3]. A large library of photopolymerized materials was screened using high-throughput technology to identify optimal substrates to support human embryonic stem cells in feeder-free conditions [6]. Orthogonal gradients in surface wettability and topography were fabricated to screen osteoblast response to biomaterial surface properties [5]. A combination of array and gradient approaches was applied to screen cell response to composition of dental composites [4]. In another study, “topochips” were designed to rapidly screen the effect of surface topography on mesenchymal stem cell proliferation and differentiation [7]. 

However, in tissue engineering, biomaterials are commonly processed into three-dimensional (3D) scaffold for tissue regeneration [8]. Cells are sensitive to topographical differences between 2D surfaces and 3D scaffolds [9,10] and studies indicate that cell response in 3D scaffolds is more representative of *in vivo* behavior than that observed in a 2D culture format [11,12,13]. Thus, there is a need to design combinatorial techniques to screen 3D tissue scaffolds. A few recent studies have utilized combinatorial 3D tissue scaffolds [14,15,16,17,18]. For example, gradient hydrogels were fabricated to screen the effect of 3D scaffold modulus on encapsulated osteoblasts [15]. Arrays of salt-leached macro-porous scaffolds were prepared to measure osteoblast response to polymer blend composition [14]. In other work, gradient and array library formats were compared for assessing osteoblast response to the mass fraction of amorphous calcium phosphate included in 3D composite salt-leached scaffolds [18]. Finally, the effect of different extracellular matrix proteins on embryonic stem cell differentiation was studied using combinatorial hydrogel scaffold libraries [17]. 

In the current work, we aimed to develop an approach for fabricating gas-foamed scaffold gradients that can be used for combinatorial screening in 3D. Previously, we developed a two-syringe pump approach for fabricating salt-leached scaffold gradients [14,18,19,20]. However, gas-foamed scaffolds have several advantages over salt-leached scaffolds. Gas-foamed scaffolds use gas bubbles to create porous polymer scaffolds [21,22,23,24]. The gas-foaming process yields scaffolds with pores that are better connected than those in salt-leached scaffolds and gas-foamed scaffolds also have stronger mechanical properties [21]. Salt-leaching also leaves a polymer skin on scaffolds that can prevent cells from penetrating the scaffold interior while the gas-foaming process yields a scaffold with uniform pore distribution and no skin [22]. Gas-foamed scaffolds support adhesion, proliferation and differentiation of osteoblasts to yield bone-like tissues [23,24].

**Figure 1:**
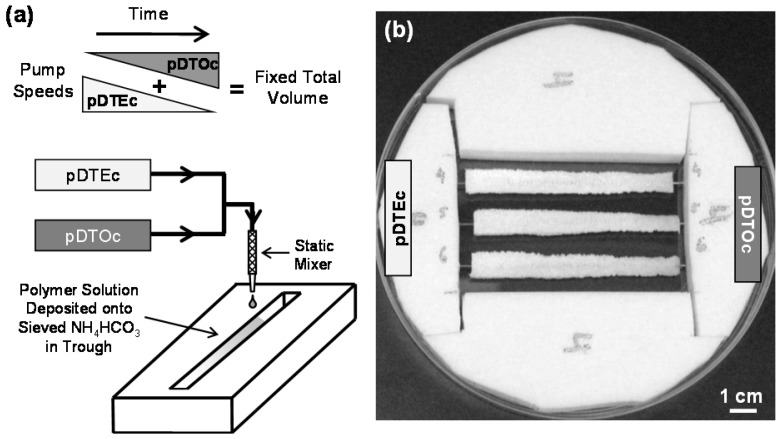
(**a**) Schematic representation of the combinatorial platform used to fabricate the gradient gas-foamed scaffold libraries. Solutions of pDTEc and pDTOc dispensed from programmed syringe pumps were mixed through a static mixer and deposited onto a trough of sieved NH_4_HCO_3_ crystals (250 μm to 425 μm); (**b**) Photograph of a Teflon rig supporting three gas-foamed gradient scaffolds in a large Petri dish. Each gradient is 75 mm long, 8 mm wide and 3 mm deep. A metal wire runs the length of each scaffold gradient enabling them to be suspended in the Petri dish for cell culture experiments.

**Figure 2:**
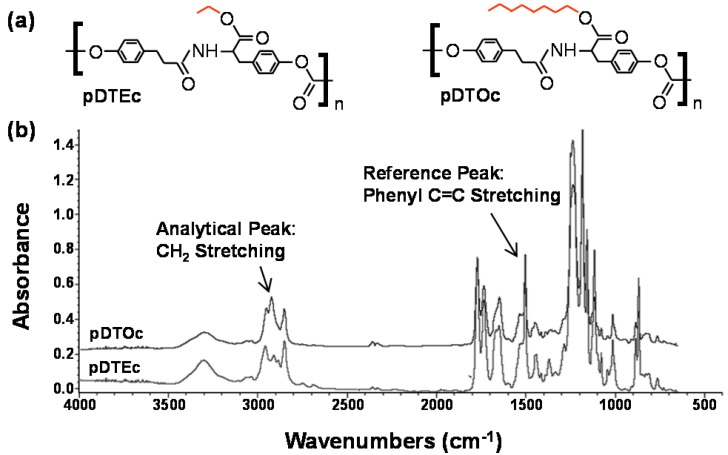
(**a**) Chemical structure of poly(desaminotyrosyl-tyrosine ethyl ester carbonate) (pDTEc) and poly(desaminotyrosyl-tyrosine octyl ester carbonate) (pDTOc); (**b**) FTIR spectra for pDTEc and pDTOc after baseline deduction and normalization to maximum absorbance.

Note: Peaks at 2,927 cm^−1^ and 1,508 cm^−1^ were chosen as the analytical and reference peak, respectively, for quantification.

A two-syringe pump approach (Figure 1) [18,19] was adapted to fabricate gas-foamed scaffold gradients. In order to demonstrate the feasibility of this approach, blends of two degradable tyrosine polycarbonates, poly(desaminotyrosyl-tyrosine ethyl ester carbonate) (pDTEc) and poly(desaminotyrosyl-tyrosine octyl ester carbonate) (pDTOc), were used to fabricate gas-foamed scaffold libraries. Tyrosine polycarbonates are being advanced for use in implantable biomedical devices such as hernia repair meshes, cardiovascular stents and bone tissue engineering scaffolds [25,26]. pDTEc and pDTOc have the same backbone but different side chains (Figure 2). These different side chains cause differences in the polymer properties including water contact angle (71° for pDTEc and 91° for pDTOc), glass transition temperature (99 °C for pDTEc and 53 °C for pDTOc), mechanics [pDTEc is brittle (4% elongation at break), while pDTOc is ductile (400% elongation at break)], degradation (pDTEc degrades faster) and biological response [14,27,28]. These differences in properties affect protein adsorption and cell responses such as cell spreading, adhesion and proliferation [27,28].

## 2. Experimental Section

### 2.1. Fabrication of Gas-Foamed Gradient Scaffolds

Poly(desaminotyrosyl-tyrosine ethyl ester carbonate) (pDTEc, relative molecular mass 183,000 g/mol) and poly(desaminotyrosyl-tyrosine octyl ester carbonate) (pDTOc, relative molecular mass 117,000 g/mol) were synthesized as described [27] and solutions of each were made in dioxane (0.1 g/mL). A two-syringe pump system [18,19] was used to fabricate gas-foamed scaffold gradients (Figure 1). Briefly, the pDTEc and pDTOc polymer solutions were loaded into syringes and placed on opposing syringe pumps. pDTEc solution (1 mL) was loaded on the left syringe pump and 2.5 mL of pDTOc solution was loaded on the right pump. The pumps were programmed for a run of 72 s such that the flow rate of the left pump (pDTEc) decreased linearly from 0.5 mL/min to zero while the flow rate of the right pump (pDTOc) increased linearly from zero to 0.5 mL/min. The two solution streams were mixed using a stainless steel static mixer (internal diameter 3.3 mm; catalog # EW-04669-54, Cole-Parmer) which mixes the polymer solutions by cutting and folding them as they flow through a series of helices. This process created effluent at a constant rate of 0.5 mL/min which changed linearly in composition from pDTEc to pDTOc as it eluted from the static mixer.

The effluent was deposited into a rectangular Teflon trough (75 mm long, 8 mm wide, 6 mm deep) containing 3 g of sieved ammonium bicarbonate (NH_4_HCO_3_, 250 μm to 425 μm). A 21 gauge orthodontic wire ran lengthwise along the center of the trough at a height of 2 mm from the bottom of the trough. The wire was designed as a “handle” for mounting the scaffolds and for picking them up. A third syringe pump was fit with a flat stage and used to translate the NH_4_HCO_3_ trough at a rate of 3.75 cm/min while the polymers solutions were deposited to create the gradient in deposition. After deposition, gradients were air dried for 30 min in a chemical hood, and then submerged in liquid nitrogen to freeze them. Frozen gradients were placed in a freezedryer overnight to remove residual solvent. Freezedried gradients were removed from the Teflon troughs and placed in warm deionized water (37 °C) for 3 h for gas-foaming of the NH_4_HCO_3_ to create pores. Gradient scaffolds were further leached 3 d in deionized water, air dried and stored in a desiccator. A total of six gradient scaffolds were prepared for this study (three for SEM and three for FTIR).

### 2.2. Characterization of Gradient Scaffolds

For characterization, gradient scaffolds were cut perpendicular to their long axis into seven equal segments (approximately 1.1 cm length per segment) using a scalpel. For scanning electron microscopy (SEM), scaffold segments were sputter-coated with gold and imaged (15 kV, Hitachi S-4700-II FE-SEM). To characterize the pore structure along the gradient, the diameter of the pores was measured in SEM images for segments 1, 4 and 7. For each segment, three SEM images were analyzed and 24 pores were sized for each segment. Scaffold segment compositions were determined by FTIR spectroscopy (NEXUS 670 FTIR spectrophotometer, Nicolet, Thermo Electron). Scaffold segments were individually dissolved in 0.1 mL dioxane and transferred onto KBr discs to record the FTIR spectra (compilation of 64 scans from 650 cm^−1^ to 4,000 cm^−1^, resolution of 4 cm^−1^). Analysis was performed with OMNIC (Version 7.2, Thermo Electron, Chicago, IL, USA). A series of solutions of known composition were prepared by mixing different ratios of pDTEc and pDTOc to prepare an FTIR calibration plot. A linear fit to the calibration plot was used to determine the composition of the unknowns. The porosity of segments 1 and 7 (pDTE-rich and pDTO-rich, respectively) from the scaffold gradients was determined as described previously [19] using the following formula: porosity = 1 − [(m/d)/v], where m is the mass of the scaffold, d is polymer density (1.2 g/cm^3^ for both pDTEc and pDTOc [5]), and v is the volume of the scaffold.

## 3. Results

A two-syringe pump approach (Figure 1) for fabricating salt-leached scaffold gradients was adapted herein to fabricate gas-foamed scaffold gradients. To demonstrate the approach, two different polymers, pDTEc and pDTOc, were used (Figure 2) to fabricate the gas-foamed scaffold libraries. Three pDTEc/pDTOC gas-foamed scaffold gradients made by the 2-syringe pump approach are shown in Figure 1b. Each gradient was 75 mm long, 8 mm wide and 3 mm deep. The scaffold gradients were mounted in a Teflon rig in a large petri dish. The wires that ran the length of the gradients are visible at the ends of the gradient and were used to suspend the scaffolds in the dish so that they could be used for cell culture experiments.

**Figure 3:**
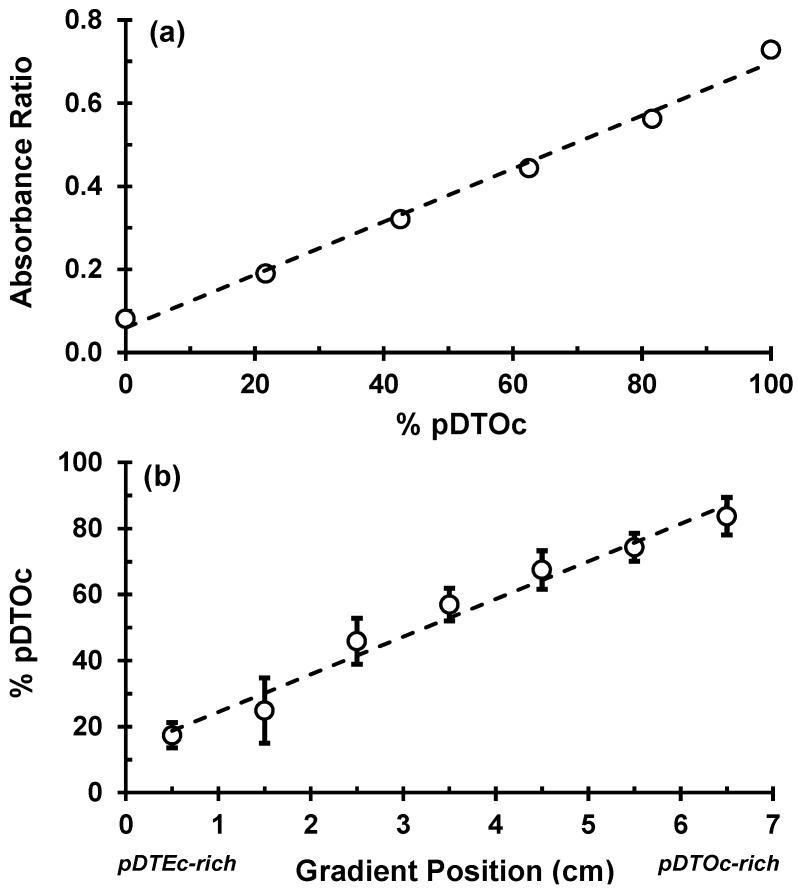
(**a**) Calibration plot of FTIR peak absorbance ratios (2,927 cm^−1^: 1,508 cm^−1^) for known control mixtures of pDTEc and pDTOc. The dashed line is a linear fit(y = 0.0064x + 0.0596) with Pearson correlation coefficient (R) of 0.99; (**b**) Plot of pDTOc content in the different segments of the gradient scaffolds (error bars are standard deviation, n = 3). The slope of the linear fit (dashed line) is significantly greater than zero (t-test, p < 0.05) with a Pearson correlation coefficient (R) of 0.98.

**Figure 4:**
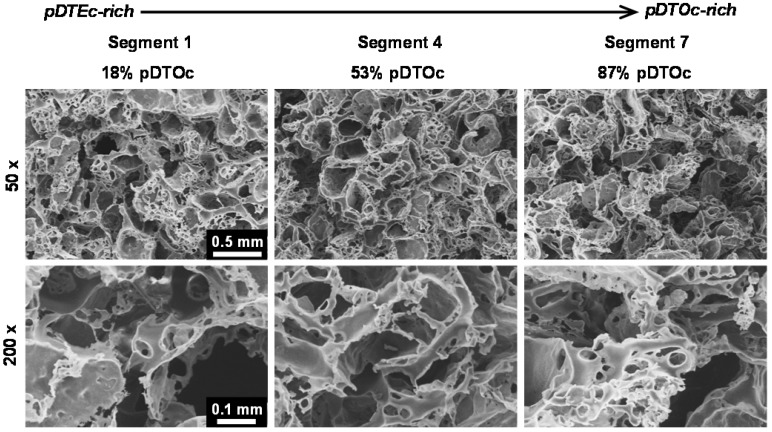
Scanning electron micrographs of the 3D gas-foamed gradient scaffolds at 50× (top row) and 200× (bottom row) magnification showing the interconnected porous structure.

FTIR was used to characterize the scaffold gradients to map composition. Control spectra for pure pDTEc and pDTOc are presented in Figure 2b. Absorbance at 1,508 cm^−1^ was chosen as the reference peak (phenyl ring (C=C) stretching) [29] since the phenyl ring structure is the same for pDTEc and pDTOc. Absorbance at 2,927 cm^−1^ was chosen as the analytical peak (CH_2_ stretching) [30] since the respective ethyl and the octyl side groups in pDTEc and pDTOc are different. An FTIR calibration curve was constructed using known blends of the two polymers (Figure 3a) so that the composition of the gradient scaffold could be assessed. Gradient scaffolds were cut into seven segments and assessed for pDTEc/pDTOc composition using the FTIR calibration plot. The mean composition of 3 gradient scaffolds ranged from 17% pDTOc at the pDTEc-rich end to 84% pDTOc at the pDTOc-rich end (Figure 3b). The average standard deviation of the mean pDTOc composition for the 7 segments of the 3 gas-foamed gradients was 6%. Previously, salt-leached scaffold gradients were fabricated and they had average standard deviations of the mean composition of 5% for Sudan IV red dye gradients [19] and 14% for gradients of amorphous calcium phosphate nanoparticles [18]. Thus, these results demonstrate that the two-syringe pump gas-foamed scaffold gradient fabrication method yields repeatable gradients that is comparable to previous gradient methodologies. The morphology of the scaffold gradients was assessed by SEM (Figure 4), which revealed a highly interconnected pore structure. The diameter of the pores were measured to be (0.21 ± 0.06) mm, (0.23 ± 0.06) mm and (0.24 ± 0.06) mm for segments 1, 4 and 7 (mean ± S.D., n = 24), respectively, and the differences were not statistically significant(*p* > 0.05). The porosity at the pDTEc-rich (segment 1) and pDTO-rich (segment 7) ends of the gradients was measured to be (97.9 ± 3.0)% (n = 5) and (96.9 ± 3.6)% (n = 6), respectively, and these values also were not statistically different (p > 0.05). These results show that the scaffold structure was similar across the gradients and that the change in pDTEc/pDTOc composition in the gradients did not affect pore structure.

## 4. Discussion

The current work establishes a two-syringe pump approach for fabricating gas-foamed scaffold gradients that can be used for combinatorial screening of cell-material interactions in 3D. Methods for salt-leached scaffolds [14,18,19], hydrogels [15,17], nanofiber scaffolds [31,32,33,34] and gas-foamed scaffolds (current work) have been described for combinatorial screening in 3D. In the salt-leaching method, the sodium chloride crystals serve as a porogen which is leached by dissolution in water. The final scaffold pores match the size and shape (cuboidal) of the starting salt crystals [10]. When gas-foaming, the ammonium bicarbonate serves as both a porogen and as a source of gas bubbles. During ammonium bicarbonate dissolution, ammonia and carbon dioxide gases are generated from the reaction with water. The release of gas bubbles provides additional interconnections between pores. The gas bubbles must squeeze out of the scaffold which changes the final pore morphology, making the pores smaller in size than the starting crystals and irregular in shape. In Kumar *et al*. [10] salt-leached and gas-foamed scaffolds were made from the same polymer (PCL) using the same sieve sizes for the NaCl and ammonium bicarbonate (250 μm to 425 μm), respectively. SEMs of these scaffolds showed that: (1) the salt-leached scaffolds had larger pores than the gas-foamed scaffolds; (2) the gas-foamed pores were more irregularly shaped than the cuboidal salt-leached pores; (3) the gas-foamed had more overall pores than the salt-leached (due to gas bubble release). The gas-foamed gradients in the current work (Figure 4) also had had irregularly shaped pores that were smaller in size (210 μm to 240 μm) than the starting ammonium bicarbonate crystals (250 μm to 425 μm).

Scaffold gradients are useful for systematically screening cell response to scaffold properties because all scaffold samples can be contained in a single culture dish to reduce well-to-well variability [18]. Scaffold gradients can also be used for engineering graded tissues [35]. For instance, ligaments and tendons join soft and hard tissues and contain gradients from cartilage to bone [36]. Scaffolds containing gradients can be used to spatially control cell function to generate tissue gradients to restore native hierarchical structure [37,38]. Herein, gas-foamed scaffolds were fabricated with a polymer composition gradient. However, the method is adaptable to any components that can be processed via liquid mixing including gradients of nanoparticles, calcium phosphate composites, encapsulated growth factors, peptide-functionalized polymers or vectors (virus, plasmid). 

## 5. Conclusions

A two-syringe pump method for fabricating gas-foamed scaffold gradients has been developed. Gradients in scaffold polymer composition were fabricated using two polymers with different properties, pDTEc and pDTOc. The new method yielded gas-foamed scaffolds with repeatable gradients in polymer composition and a consistent pore structure. This new approach for fabricating gas-foamed scaffold gradients can be used for combinatorial screening of cell-material interactionsin 3D. 
